# *Cronobacter* Species Contamination of Powdered Infant Formula and the Implications for Neonatal Health

**DOI:** 10.3389/fped.2015.00056

**Published:** 2015-07-02

**Authors:** Gautam Kalyantanda, Lyudmila Shumyak, Lennox Kenneth Archibald

**Affiliations:** ^1^Division of Infectious Diseases, College of Medicine, University of Florida, Gainesville, FL, USA; ^2^Malcom Randall VA Medical Center, Gainesville, FL, USA

**Keywords:** *Cronobacter*, *Enterobacter*, *sakazakii*, powdered infant formula, neonatal mortality, necrotizing enterocolitis, neonatal sepsis, neonatal meningitis

## Abstract

*Cronobacter* is a class of *Enterobacteriaceae* that cause infections in neonates, especially those born prematurely. Over 90% of these infections have been linked epidemiologically to powdered infant formula (PIF). Contamination of PIF can occur at manufacture, reconstitution, or storage of reconstituted product. Intrinsic properties that enable *Cronobacter* to cause disease include resistance to heat, ultraviolet radiation, oxygen radicals, stomach acids, and pasteurization; an ability to utilize sialic acid (a nutrition additive to PIF that facilitates the organism’s growth and survival), and an exceptional affinity for biofilms in enteral feeding tubes. As part of ongoing endeavors to reduce the incidence of neonatal PIF-associated *Cronobacter* infections, the World Health Organization and the US Food and Drug Administration have established guidelines for PIF production, preparation for infant feeding, and storage of reconstituted product.

## Introduction

*Cronobacter* spp. belong to the *Enterobacteriaceae* family and are oxidase-negative, facultative anaerobic, Gram-negative bacilli with circumferentially arranged flagellae that render the microorganisms their motility (1, 2). Before 2008, the genus *Cronobacter* spp. was referred to singularly as *Enterobacter sakazakii* in the medical literature until it was noted that there was significant variability among the various *E. sakazakii* isolates. This finding led to their reclassification to the *Cronobacter* genus (3).

The natural habitats of *Cronobacter* spp. are predominantly plant and organic material. The pathogen has been isolated from various food sources – cheese, meat, milk powder, powdered infant formula (PIF), infant weaning foods, chocolate, dry cereals, potato flour, fresh and dried herbs and spices, and pasta (3, 4). The microorganism has also been isolated from the nasopharynx and gastrointestinal tract of humans, the home environment (e.g., house dust, vacuum cleaner collection bags, and kitchen utensils), hospital environments, flies and rodents, and in PIF manufacturing facilities (4). However, when compared with other members of the *Enterobacteriaceae* family or with non-fermentative Gram-negative bacilli (e.g., *Pseudomonas* spp. or *Acinetobacter* spp.), *Cronobacter* spp. have rarely been implicated as a significant cause of healthcare-associated infections or outbreaks (5).

## Epidemiology of PIF-Associated *Cronobacter* Infections

*Cronobacter* spp. are associated with systemic infection, meningitis, and necrotizing enterocolitis in premature infants. The incidence of infection in the US is reported to be >9 infections per 100,000 neonates of very-low-birth-weight (4, 6, 7). There are much fewer reported cases of *C. sakazakii* infections in adults with most occurring in persons with malignancies and other debilitating conditions (8, 9).

In a comprehensive review of *Cronobacter* infections documented in Centers for Disease Control and Prevention (CDC), US Food and Drug Administration (FDA), and World Health Organization (WHO) records, peer-reviewed publications, and personal communications from across the globe for the period 1958 through 2010, Jason confirmed the following: (a) neonates were predominantly affected (99% of case-infants <3 months of age); (b) 90% of case-infants had received PIF beforehand; and (c) >50% of the infections ascertained during 2004–2014 occurred in the home setting (10).

Friedemann studied >100 neonatal *Cronobacter* spp. infections between 2000 and 2008 and documented a 42% mortality among neonates with *Cronobacter* spp. meningitis (11). Among *C. sakazakii* infant case consultations conducted by CDC during 1998–2005, 92% of infants for whom information on feeding practices were available had received a PIF product (12).

Before 1994, *C. sakazakii* was not recognized as a significant cause of neonatal infection and its association with PIF had not yet been established. Since then, a myriad of published reports and studies have identified contaminated PIF as the main risk factor for neonatal *Cronobacter* spp. infections (10, 12–21). *C. sakazakii* strains appear to elicit the type 2 immune response, which is known to be inefficient in fighting intracellular infections, and would explain neonatal susceptibility to unique *Cronobacter* strains (22).

Contamination with *Cronobacter* spp. is a global issue. For example, *Cronobacter* spp. have been isolated from PIF in Korea, from salads, vegetables, minced meat, and produce in Thailand, and from feed and grain samples in Australian farms (23–27).

## Why PIF is at risk for *Cronobacter* spp. Contamination?

Intrinsic contamination of PIF can occur at any stage during manufacture at the factory before distribution of product for retail; extrinsic contamination of product can occur after the factory container is first opened by the user; at any stage of reconstitution through the use of contaminated water, utensils, work surfaces; at the time of feeding (e.g., using contaminated feeding bottles or enteral tubing with existing biofilm); or because of inappropriate storage conditions (e.g., poor refrigeration or storage for too long at room temperature) (7). Underlying all the occurrences (whether at the site of manufacture, or during reconstitution at the hospital, or the home) is unsatisfactory or non-adherence to appropriate infection control practices and procedures.

Because reconstitution of PIF in hot water significantly reduces *Cronobacter* bioburden, it is believed that the manufacturing phase after pasteurization is the juncture where intrinsic contamination frequently occurs. Contamination of PIF can occur if the plant-derived PIF ingredients are not properly pasteurized with prior heat treatment (28, 29). The importance of having a validated pasteurization process for PIF manufacture is underscored by the fact that bioburden levels in contaminated PIF product are generally low (never > 1cfu/gram) (3, 4, 21).

Powdered infant formula contamination can occur during the remaining stages in the production process, especially during phases close to the packing of the final product (30, 31). Proudy et al. found that the majority (78%) of isolates were recovered from processing areas (surfaces around dryers and blenders); 12% from ingredients and 10% from final product (31).

Intrinsic properties and survival mechanisms that enable *Cronobacter* spp. to end up as a PIF contaminant include resistance to desiccation and osmotic stresses (3). The microorganism has a heteropolysaccharide capsule that is resistant to osmotic desiccation. *Cronobacter* strains can therefore survive for relatively long periods (often >2 years) in the desiccated state in PIF and other powdered foods. By contrast, non-capsulated strains have shorter survival spans and are relatively less pathogenic (3).

*Cronobacter* spp. are also tolerant to acids, a property that renders them resistant to the low pH environment in the human stomach; some species produce an *enterotoxin* that is heat-stable and able to survive pasteurization of PIF (16). The microorganism also generates an *endotoxin* lipopolysaccharide that facilitates the translocation of the bacteria across the intestinal wall and across the blood-brain barrier (4). This endotoxin is heat-stable and able to remain viable for long periods in reconstituted PIF (16).

Sialic acid is an additive to PIF during manufacture because of its reported ability to foster healthy brain development in the neonate (4). *C. sakazakii* is unique within the *Cronobacter* genus in that it can utilize sialic acid for growth. This property enables *C. sakazakii* to remain viable in PIF brands that are both contaminated with the microorganism and fortified with sialic acid.

*Cronobacter* spp. bacterial cells tend to attach more readily to hydrophobic surfaces, such as Teflon and plastics, than to hydrophilic surfaces, such as glass and metals (16). Thus, if PIF is contaminated with *Cronobacter* spp. during manufacture, the reconstitution process basically allows formation of a medium that will facilitate bacterial growth, especially if containers and feeding bottles are made of Teflon or plastic materials.

*Cronobacter* spp. can form biofilms, which render them resistant to high-level disinfection (1). The potential for biofilms to play a key role in the chain of transmission is underscored by the observation that, compared with other *Enterobacteriaceae*, *Cronobacter* spp. tend to have the highest biofilm cell density. Several outbreaks of *Cronobacter* infections abroad and in the US have involved infants who were fed reconstituted PIF via enteral feeding tubes over a period of several hours at ambient temperature (2, 4, 21). Enteral feeding tubes with biofilms may act as loci for the attachment and multiplication of *Cronobacter* spp. Thus, organisms will enter the stomach of the neonate whatever type of infusate or feed bolus passes through an enteral tube with a colonized biofilm. This is the likely scenario in the neonatal critical care setting rather than the home.

Multiplication of *Cronobacter* in the syringe used for introducing reconstituted PIF through the enteral feeding tubes may also cause large numbers of the microorganism to be introduced into the infant, especially if the filled syringe was stored at room temperature after reconstitution (4). For enteral tubes with aged, colonized biofilms, cells shed from the biofilm may survive passage through the neonate’s stomach and contribute to the onset of infection (4, 32, 33).

## Environmental and Product Testing Recommendations

Food and Drug Administration instituted a method that combines a polymerase chain reaction (PCR) assay in combination with two chromogenic agars that reduce the time needed for identifying the microorganism from 5 to 3 days (23). FDA’s end-product testing protocol is based on the presumption that the pathogen is uniformly distributed througout PIF batches deemed to be contaminated with *Cronobacter*. In fact, *Cronobacter* spp. tend to be non-homogeneously distributed in PIF by clumping to form spatial clusters in end-product (34). This property limits the utility of random sampling of PIF batches for quality assurance or public health surveillance.

The FDA method recommended for *Cronobacter* ascertainment has since been revised to combine real-time PCR, chromogenic agars, and RAPID ID 32E biochemical tests for isolation and detection of *Cronobacter* within 24–48 h (35, 36). For epidemiologic characterization and analyses, PFGE with two restriction enzymes is currently the most commonly used molecular method (37).

## Global Surveillance of *Cronobacter* spp. Infections

Most serious cases of *Cronobacter* neonatal infections during the past 30 years have been caused by strains with a highly stable molecular sequence profile (*C. sakazakii* sequence type 4 or ST4) (23, 38). These four distinct pulse-types have been implicated in outbreaks associated with necrotizing enterocolitis, septicemia, or meningitis. Phenotyping schemes and 16S rDNA sequence analyses have identified a total of 16 biotypes among *Cronobacter* spp. (39–44).

However, only one specific pulse-type appears to be associated with significant mortality and the ability to penetrate intestinal and blood-brain barrier cells (40, 41). At the present time, molecular typing methods are being increasingly used as a faster and more reliable tool to identify, classify, study genomic diversity, and track sources of infection, and have largely replaced phenotyping methods in the characterization of *Cronobacter* spp. (2, 44).

In 2004, the WHO instituted a molecular typing scheme to enable the international control of the *Cronobacter* spp. infections. Baldwin and colleagues surmised that sequencing data from multiple gene loci rather than one locus might be better phylogenetic markers and would enable better discrimination between strains compared with 16S rDNA sequencing (39). They established a comprehensive genotyping scheme based on multi-locus sequence typing (MLST) for *Cronobacter* spp. The scheme is based on seven gene loci with specific functions, including DNA repair, replication, amino acid biosynthesis, and wide distribution across the chromosome. This led to the establishment of the *Cronobacter* PubMLST genome and sequence definition database (http://pubmlst.org/Cronobacter/) containing over 1000 isolates and detailed data on specific clonal lineages linked to neonatal meningitis and adult infections (44, 45). This database is a central, open access, reliable, sequence-based source for researchers. It enables integration of clinical, epidemiologic, microbiologic, and molecular typing data and retrospective analyses of historic cases and outbreaks. By applying the MLST scheme to over 1000 strains, Forsythe and colleagues identified 298 definable sequence types. Of these 298 strains, only one – *C. sakazakii* clonal sequence type 4 – again was found to be associated with neonatal meningitis (45). This strain is thought to be a stable clone because it has been identified in seven countries for over 50 years (46).

## Prevention of PIF-Associated *Cronobacte*r Infections in Neonates

Though there are no specific testing recommendations, the Food and Agriculture Organization/World Health Organization (FAO/WHO) established a list of recommendations for hospitals and homes regarding PIF preparation for infants during the first 2 months of life, when the risk of infection is highest (47, 48). These recommendations are summarized as follows:

(i) PIF should be reconstituted with hot water (>70°C) to reduce the *Cronobacter* spp. vegetative concentration.
(ii) Utensils for formula preparation must be disinfected.
(iii) Reconstituted PIF should be refrigerated at 4°C if not used immediately, with disposal if not used within 24 h of preparation.
(iv) Reconstituted PIF should be kept at room temperature for a maximum of 4 h if not used immediately, and should never be kept warm in bottle heaters or thermos flasks.
(v) If storage is deemed necessary or unavoidable, it should be at temperatures <5°C.
(vi) Hospitals need to establish protocols, in the event of product recall, so that product batch follow-up and accurate data aggregation could be carried out.

Maintenance of enteral feeding tubes in neonates is of paramount importance in the efforts to prevent healthcare-associated adverse events, especially in neonatal intensive care units. Neonatal critical care specialists, infection control preventionists, and hospital epidemiologists need to appreciate the risks associated with biofilm formation in enteral feeding tubes used for neonatal nutrition. Healthcare administrators need to ensure that PIF procured for feeding high-risk infants in their respective facilities is sterile or has undergone a validated sterilization process (23).

Regulatory standards for infant food manufacturers need to be improved. Yan et al. recommend effective environmental monitoring programs, good manufacturing practice guidelines, and procedures and hazard analysis and critical control point (HACCP) systems to control the risk of microbiological contamination along the entire production chain, from the starting raw materials, throughout the entire process, until the final product is ready for distribution (23). PIF manufacturers also need to improve the labeling of PIF products and communication with consumers to enhance awareness of the correct methods for reconstituting PIF products. Education of healthcare professionals to help them provide quality training for parents and caregivers in PIF preparation, handling, and storage is particularly important for economically less-developed countries, where PIF use is relatively common.

In conclusion, adherence to regulatory standards by PIF producers and to FAO/WHO guidelines for PIF reconstitution and storage by consumers, and continuing surveillance and educational activities by CDC, FDA, and WHO are essential for the prevention of PIF-associated *Cronobacter* infections and other opportunistic pathogens in neonates.

## Conflict of Interest Statement

None of the authors have any conflict of interest or financial disclosures. In addition, none of the authors received any financial remuneration for writing this manuscript. Dr. Lennox Kenneth Archibald is the Hospital Epidemiologist at the Malcom Randall Veterans Administration Medical Center, a US Government medical facility for military veterans. The opinions in this paper are solely those of the authors and do not reflect the opinion of the US Government.
